# The Royal Wedding

**Published:** 1893-07-01

**Authors:** 


### The Hospital

AN INSTITUTIONAL JOURNAL OF

**Science, Medicine, Nursing, and Philanthropy.**

*For Hospitals, Asylums, Infirmaries, Dispensaries, Medical Practitioners, Students, Nurses, and the Charitable Public.*

VOL. XIV.—No. 353.] JULY 1, 1893.

### THE ROYAL WEDDING.

THERE are two classes of persons connected with hospitals and the medical profession who have the best of reasons for wishing to pronounce, in Lord Tennyson's phrase, "A Full God Bless You," on the Royal pair who are to be united in holy wedlock within the next few days. Those two classes are the patients and the nurses. Undoubtedly medical men and hospital officials of all sorts and conditions will be as prompt and whole-hearted in their loyalty as the patients and the nurses; but, as a class, they can hardly feel quite the same feeling of deep personal obligation and affection. Many of our English monarchs have won immortality in story and in song. We have but to pronounce the names of Alfred the Great, Elizabeth the Warrior Queen, and Victoria the Mother of her People. The Queen is still with us, and so far as human eye can penetrate has many years of happiness and high duty yet before her. The Prince and Princess of Wales may be called in a special sense the Patrons and Protectors of hospitals and the sick. There is abundant evidence to show that our Sailor Prince and his chosen bride will follow in the same footsteps. The Duke of York is already president of one or more hospitals; and it is said that the dowry which the Lord Mayor and the provincial mayors and magnates wish to bestow upon the Royal pair is designed by his Royal Highness for the founding and endowment of a convalescent home.

This is an age which demands great things of Royal persons, but it does not give them great opportunities. At least, it does not give them opportunities which have hitherto been looked upon as great. Not so very many years ago the service of hospitals and the care of the sick were looked upon as the proper duties of women and priests. Physicians and surgeons in those by no means remote times were regarded by the bluff and busy Englishman as of almost less importance than either women or priests. We have changed all that. It has been changed to a large extent during the reign of Queen Victoria. The Queen herself and the Prince and Princess of Wales have done more to elevate the work of hospitals in the esteem of all classes of the people than almost all the other forces of elevation put together. Time was, and quite recently, when a fighter of battles was the god of the nation's idolatry. To day the man who shall discover a *bona fide* cure for cancer or consumption will be acknowledged the hero of the world.

Here is a vast change in the temper and judgment of civilised man. It is a change which is profound and indubitable. It has been largely made during the present reign; it has been led and influenced by the present Royal family. The fact is undeniable that the Prince and Princess of Wales have lent themselves to every pageant of peace and charity without stint, and with the most steady and consistent goodwill. What does the British nation desire most? In what does it find its greatest happiness? Though the most warlike of all the nations it desires peace above all things, and the noble conquests of peace; though the least sentimental and demonstrative of peoples, it loves and values goodwill and good deeds as no other nation has ever yet loved and valued them. If England could have her way she would never fight; she would conquer the world by her practical capacity for bringing order out of every kind of civilised and uncivilised chaos, and by making life easier, happier, and more prosperous in every clime.

The essential characteristic of the modern Briton is sense; the capacity for seeing straight and seeing true; the practical discernment to know what to do, to know what is demanded of him by the rapidly moving times. Judged by this standard, who is so essentially British as Her Majesty the Queen? And who but the Prince of Wales, by universal consent, stands next to Her Majesty in this distinguishing characteristic? May we not go a step further and say that the Duke of York, so far as he is yet known by the people, has all the straightforward English manliness of character which has made the Prince of Wales the most universally and enduringly popular prince of his times?

As in some sort representing hospitals and the sick we stand among the practicalities of our peculiar times; and, as practical, we feel compelled to speak as we have found. This is what we have found—that the sick poor of London and Great Britain have had no friends more sincere, more deeply anxious to be of service according to their capacity, more discriminating and more consistently helpful than the members of the Royal family. They have not confined themselves to the ornamental part of hospital work. Indeed, we have good reason for saying that the ornamental is the part they care for least. Money, and time, and personal influence have always been at disposal for worthy practical objects. We may be pardoned for recurring to one object in particular to which the Prince and Princess of Wales devoted an amount of anxious thought and labour, and of money too, the extent of which will never be known. We refer to the Royal National Pension Fund. The nurses of that fund, and all connected with it, owe much of their present success and their future security to the steady and persevering determination of the Prince and Princess of Wales to carry a great practical object to a successful termination. All nurses are, in fact, under the deepest obligations to the Royal family. Her Royal Highness Princess Christian may be called the founder and establisher of the Royal British Nurses Association. Though we have often felt it our duty to fight on the opposite side, we have always had a sincere admiration for the courage and unyielding perseverance which resolved to carry the citadel, if need were, against all the world. Opponents sometimes feel greater respect for one another than fellow-soldiers, and we confess to a genuine admiration for the Princess Christian's truly Royal fighting qualities.

The Duke of York and the Princess May inherit great traditions, and they look forward to a great future. We have an excellent warrant for assurance that their future will be worthy of their traditions and of themselves. That warrant is the undoubted strength and simplicity of their characters. We know nothing of either of them but what is English and good; we know much of them both which endears them to that British heart which is at once simple and strong. We are the most progressive, and at the same time the most conservative of all peoples. We move forward steadily in practice, not by abstract theorisings; and as far as is possible we carry our past with us. But this surely is right; and it is as beautiful as it is British and sensible. Why should we cut ourselves off from our past? It has been great and glorious without parallel, and its ennobling influence may make our future great and glorious too. It is a splendid destiny to look forward to, to be King and Queen of so free and so great a people. But the task, though great, is easy. Because we are a free people we love rule and authority, and every one of us is personally concerned in their conservation. Our Kings and our Queens represent not only the majesty of monarchy, but the greater majesty of the nation and its liberties and laws.

This marriage of a British Prince with a British Princess is an occasion of personal and heartfelt satisfaction to the whole people. The assurance of its fitness is absolute; the certainty of its happiness is universal. Speaking for hospitals, for their well-cared for and happy patients, and for all the brave and strenuous men and women who devote their lives to the great battle of peace, tenderness, and amelioration, we desire respectfully and loyally to offer our congratulations. May the heavens smile, may the nations of the earth rejoice with us, and may a long life of peace and noble deeds bless this happy union!

